# Clinicopathological Features of Right vs. Left Colorectal Carcinomas: Do the Differences Really Matter?

**DOI:** 10.3390/life16020242

**Published:** 2026-02-02

**Authors:** Aura Jurescu, Alis Dema, Sorina Tăban, Robert Barna, Adrian Ovidiu Văduva, Octavia Vița, Remus Cornea, Dorela-Codruța Lăzureanu, Anca Mureșan, Mărioara Cornianu, Bianca Natarâș, Ioana Hurmuz, Adelina Vidac, Sorin Dema

**Affiliations:** 1 Department of Microscopic Morphology—Anatomic Pathology, ANAPATMOL Research Center, “Victor Babeș” University of Medicine and Pharmacy, Eftimie Murgu Square No. 2, 300041 Timișoara, Romania; jurescu.aura@umft.ro (A.J.); robert.barna@umft.ro (R.B.); vaduva.adrian@umft.ro (A.O.V.); vita.octavia@umft.ro (O.V.); cornea.remus@umft.ro (R.C.); lazureanu.dorela@umft.ro (D.-C.L.); muresan.anca@umft.ro (A.M.); cornianu.marioara@umft.ro (M.C.); ioana.hurmuz@umft.ro (I.H.);; 2 Department of Pathology, “Pius Brinzeu” County Emergency Clinical Hospital, Bulevardul Liviu Rebreanu No. 156, 300723 Timișoara, Romania; 3 Doctoral School, “Victor Babeş” University of Medicine and Pharmacy, Eftimie Murgu Square No. 2, 300041 Timișoara, Romania; 4 Department of Radiotherapy, City Emergency Clinical Hospital, Victor Babeş No. 22, 300254 Timișoara, Romania; dema.sorin@umft.ro; 5 Department of Oncology, “Victor Babeş” University of Medicine and Pharmacy, Eftimie Murgu Square No. 2, 300041 Timișoara, Romania

**Keywords:** colorectal cancer, clinical–pathological parameters, tumor location, RCC, LCC, rectal carcinomas

## Abstract

Background and objectives: Colorectal cancer (CRC) presents a variety of molecular and pathological characteristics due to its location in the large intestine, which influences its management and prognosis. We aimed to evaluate the clinicopathological disparities between right colon (RCC), left colon (LCC), and rectal carcinomas. Materials and methods: A retrospective observational study was conducted to examine consecutive cases of colorectal carcinomas diagnosed at the “Pius Brinzeu” County Emergency Clinical Hospital (PBCECEHT), Romania. The clinicopathological characteristics and metastatic spread were analyzed by the site of the malignant tumor (right colon, left colon, or rectum). Results: A total of 1812 patients met the inclusion criteria, predominantly males (57.95%). Patients with RCC had an almost equal distribution between sexes, while patients with LCC and rectal carcinomas were more frequently males (*p* < 0.0001). RCC tumors were mostly high-grade (*p* < 0.0001), deeply invasive (*p* < 0.0001), and mucinous (*p* = 0.0109), with lymphovascular invasion and distant metastases. Conclusions: We observed different clinicopathological characteristics of CRC depending on the site of origin. We emphasize that tumor location is a parameter worth considering in CRC patients, both in therapeutic management and in future clinical trials.

## 1. Introduction

The last decade has seen significant progress in the early detection, identification of prognostic markers, and therapeutic management of colorectal cancer (CRC). Still, it remains one of the most common types of cancer [1,2,3,4]. According to the *Globocan Cancer Observatory*, substantial increases in CRC incidence and mortality are expected, with 85.5% more new cases and 103.0% more deaths by 2050 [5,6,7,8]. In 2022, globally, taking into account both sexes, there were 1,926,425 (9.6%) new cases and 904,019 (9.3%) deaths from CRC [3,9]. In Romania, CRC ranked first in incidence, with 13,541 (12.9%) cases, and second in mortality, with 7381 (13.1%) deaths from this neoplasm in 2022 [10].

Nowadays, CRC is no longer considered a single entity, as clinical and pathological differences have been described between proximal and distal colon carcinomas [11,12,13]. The reason for these differences is not fully understood. According to the literature, the disparities between right-sided colon carcinomas (RCC) and left-sided colon carcinomas (LCC) are likely due to their different embryological origins, anatomical and vascular differences, and genetic mutations associated with distinct carcinogenesis pathways [14,15]. Based on the embryological origin of the affected colon, some authors have suggested that patients be grouped by site of primary tumors: patients with proximal colon carcinomas (RCC—tumors of the cecum, ascending colon, hepatic flexure, transverse colon) and patients with distal colon carcinomas (LCC—tumors of splenic flexure, descending colon, sigmoid colon, rectosigmoid junction) [16,17,18]. LCC is more often associated with chromosomal instability, whereas RCC is more frequently characterized by microsatellite instability (MSI), BRAF mutations, and a methylated CpG island phenotype [12,19]. Most CRCs are LCCs or rectal tumors, but a possible change in the distribution of CRC is reported, with a trend towards a decrease in the incidence of distal colon tumors [13,19]. This is mainly due to advances in diagnostic techniques and the effectiveness of screening programs. LCCs are more common in men, and an increasing incidence is reported in patients less than 50 years of age [20]. In contrast, RCCs are relatively more common in women and in elderly patients [13,21]. RCCs also present at a more advanced stage of the disease, and surgical complications are more common in these patients [13,22]. According to recent studies, the prognosis of advanced RCC is poorer than that of LCC, but this may not hold true at every stage of the disease [13,19]. However, patients with metastatic RCC had lower response rates to anti-EGFR therapy and chemotherapy compared to those with LCC [21,23]. Nevertheless, the CRC’s location is not yet a characteristic to be considered in therapeutic decisions (adjuvant or palliative chemotherapy).

### Study Aim and Objectives

The aim of our study was to assess clinicopathological differences among RCC, LCC, and rectal carcinomas by analyzing histopathological reports of patients with CRC diagnosed on surgical resection specimens. We set the following specific objectives:

- To create a database and evaluate the main clinico-morphological characteristics of colorectal tumors through a retrospective study spanning a 10-year period, which included CRC cases diagnosed at the “Pius Brinzeu” County Emergency Clinical Hospital, Timisoara (PBCECHT);
- To evaluate the classic prognostic factors such as age, sex, histological type, differentiation grade (G), tumor extension (pT parameter), nodal status (pN parameter), distant metastases (pM parameter), and lymphovascular invasion (LVI), depending on the location of the primary tumors.

## 2. Materials and Methods

Cases of colorectal carcinomas diagnosed on surgical resection specimens with regional lymphadenectomy from patients with CRC diagnosed in the Surgery Clinics of the “Pius Brânzeu” County Emergency Clinical Hospital in Timișoara (PBCECHT) were followed for 10 years (2009–2018).

### 2.1. Ethics Statement

The study complied with the principles of the Declaration of Helsinki and received approval from the Ethics Committee of PBCECHT (Approval no. 460/15 April 2024). According to Romanian legislation, each patient gave informed consent upon admission, allowing research studies and photography of tissues or organs for didactic/scientific purposes.

### 2.2. Data Collection

The cases were selected after analyzing histopathological reports in the Pathology Department database at PBCECHT.

*Inclusion criteria*:

- Consecutive cases of colorectal carcinomas diagnosed following histopathological processing of radical surgical resection specimens with regional lymphadenectomy.

The *exclusion criteria*:

- Patients with a histological type of CRC other than carcinomas.
- Patients with synchronous or metachronous CRC tumors.
- Patients who received neoadjuvant radiochemotherapy treatment before surgery.
- Patients with tumor recurrences.

### 2.3. Histopathological Examination

Tissue fragments taken from surgical resection specimens for histological analysis were processed by paraffin embedding. Tissue sections with a thickness of 3–4 µm, resulting from paraffin blocks, were stained by the usual hematoxylin-eosin (HE) method.

#### 2.3.1. WHO Tumor Classification and AJCC Tumor Staging

The tumor histological subtype and grade were assessed using the *WHO Classification of Digestive System Tumors* [24]. The staging parameters were evaluated according to the pTNM system in the *AJCC/UICC Cancer Staging Manual*, developed by the American Joint Committee on Cancer (AJCC) and the International Union for Cancer Control (UICC) (editions available at the time of diagnosis) [25,26].

#### 2.3.2. Recorded Data

The following clinicopathological parameters obtained from pathology reports have been entered into the Excel table.

- Age: patients aged ≤60 years and >60 years, respectively.
- Patient sex: F (female) and M (male).
- The location of the CRC was considered as follows:
  - tumors belonging to the cecum, ascending colon, hepatic flexure of the colon, and transverse colon were considered tumors of the right colon;
  - tumors located at the splenic flexure of the colon, descending colon, sigmoid, and recto-sigmoid junction were considered tumors of the left colon;
  - tumors of the rectum.
- According to WHO [24,27] criteria, the histological types identified were: ADK NOS (classic, conventional type adenocarcinoma), mucinous adenocarcinoma, signet-ring cell carcinoma, and medullary carcinoma. The following were analyzed:
  - cases of ADK NOS, this category also includes cases of adenocarcinoma with a mucinous component (where intracellular and extracellular mucin does not exceed 50% of the tumor area);
  - mucinous adenocarcinomas, cases in which extracellular mucin-containing malignant cell clusters were identified in more than 50% of the tumor;
  - other histological types (signet ring cell carcinomas, medullary carcinomas).
- The degree of tumor differentiation (G) was assessed according to the percentage of gland formation:
  - low-grade carcinomas (G1–G2, ≥50% gland formation),
  - high-grade carcinomas (G3–G4, 0–49% gland formation) [24,27].
- Tumoral extension into the intestinal wall (pT):
  - tumors with invasion into the mucosa (pT1);
  - tumors invading the submucosa (pT2);
  - tumors invading the subserosal/subserosal adipose tissue (pT3);
  - tumors invading or exceeding the serosa (pT4) [25,26].
- Lymph node status (pN) was interpreted as follows:
  - absence of nodal metastases (pN0);
  - presence of metastases in 1 to 3 lymph nodes (pN1), or in more than 4 lymph nodes (pN2) [25,26].
- Metastases present in lymph nodes located distant from the primary tumor or in other organs (pM): only pathologically documented metastases (pM1) were considered.
- Lymphovascular invasion (LVI) was considered as absent/present (LV0/LV1).

We mention that in one of our articles, this group of patients was partially described from the perspective of analyzing demographic data and clinicopathological parameters [20].

### 2.4. Statistical Analysis

The statistical analysis of the collected parameters was performed using the GraphPad Prism software, v8.2 (GraphPad Software Inc., San Diego, CA, USA) and Microsoft Excel 2010 (Microsoft Corp., Redmond, WA, USA). Numbers and percentages were used to present the data. To analyze the differences between categorical variables, we employed the exact Chi-squared or Fisher test. Statistical significance was indicated by a *p*-value < 0.05.

## 3. Results

### 3.1. Histopathology and Patient Characteristics

Based on the established criteria, we included 1812 cases in the study. Patients ranged in age from 24 to 93 years, with an average age of 65.59 years. Table 1, Table 2 and Table 3 show the patients’ clinicopathological characteristics.

**Figure 1:**
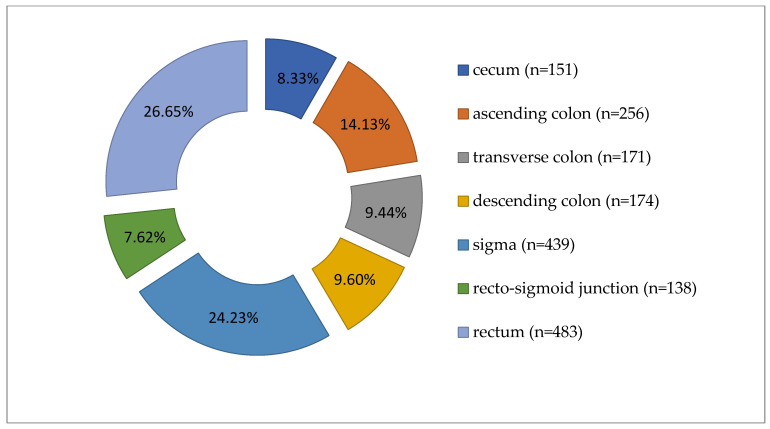
Distribution of carcinomas cases according to tumor location along the large intestine (*n* = 1812).

Colorectal carcinomas were more prevalent in males (1050 cases, 57.95%) than in females (762 cases, 42.05%). Tumors were diagnosed predominantly in patients over 60 years of age (*n* = 1261, 69.59%). Regarding the location of tumors along the segments of the large intestine, the distribution of cases was as follows: cecum (151 cases), ascending colon (256 cases), transverse colon (171 cases), descending colon (174 cases), sigma (439 cases), recto- sigmoid junction (138 cases), and rectum (483 cases), as shown in Figure 1.

Grouping the cases, we noted 578 tumors (31.90%) in the right colon, 751 (41.45%) in the left colon, and 483 (26.65%) in the rectum (Table 1, Figure 1). The average age was 65.96 years for LCC patients, 66.18 years for RCC, and 64.31 years for rectal carcinoma. Most cases were ADK NOS (*n* = 1612, 88.96%), with 191 cases (10.54%) mucinous ADK. Other histological types, such as signet ring cell carcinomas (*n* = 7) and medullary carcinomas (*n* = 2), made up only 0.50% of 1812 cases. Low-grade malignancy tumors (G1-G2) were found in 1519 cases (83.83%). Tumors infiltrated the intestinal wall beyond the muscularis propria (pT3-pT4) in 86.15% of cases (*n* = 1561). Lymph node metastases (pN1-pN2) were present in 896 cases (49.45%). Distant metastases (pM1) were pathologically documented in 112 cases (6.18%), and lymphovascular invasion in 853 cases (47.08%) (see Table 1 and Table 2).

### 3.2. Evaluation of Clinicopathological Parameters According to Tumor Location

The analysis of associations between clinicopathological parameters and primary tumor location is shown in Table 2 and Table 3.

**Figure 2:**
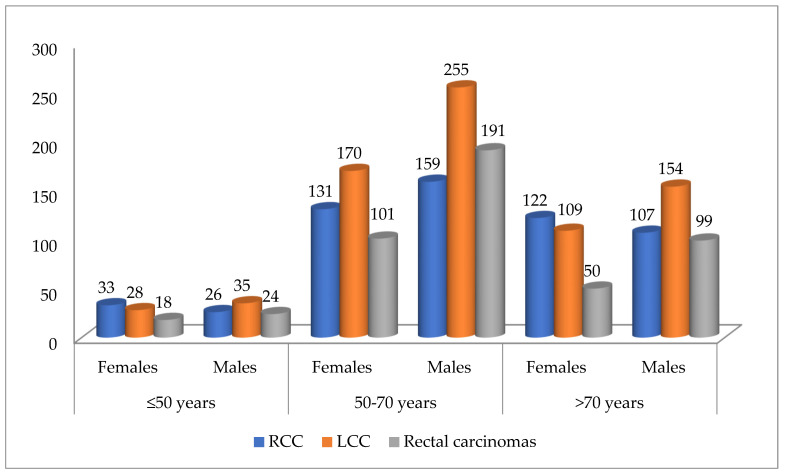
Distribution of cases according to tumor location, sex, and age intervals (*n* = 1812). RCC—right colon carcinomas. LCC—left colon carcinomas.

**Figure 3:**
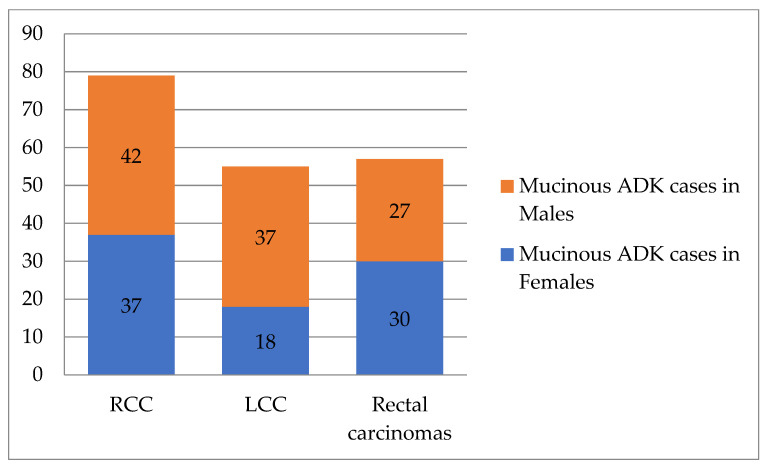
Distribution of mucinous ADK cases according to tumor location and patient’s sex (*n* = 191). RCC—right colon carcinomas. LCC—left colon carcinomas. ADK—adenocarcinomas.

- Regarding *patients’ sex*, in women (*n* = 762), right colon involvement was most common (*n* = 286, 49.48%), followed by the left colon (*n* = 307, 40.88%). In men (*n* = 1050), left colon tumors (*n* = 444, 59.12%) and rectal tumors (*n* = 314, 65.01%) were more frequent. The statistical differences were highly significant (Chi-Square test: *p* < 0.0001, Fisher’s exact test: *p* < 0.0001, OR = 1.56, 95%CI: 1.278 to 1.904). See Table 2 and Table 3.
- When analyzing tumor topography by *patients’age*, we found that in patients aged ≤60 years (*n* = 551), left colon carcinomas (*n* = 223) were more common. This was followed by rectal tumors (*n* = 169) and right colon tumors (*n* = 159). For patients aged >60 years (*n* = 1261), left colon tumors (*n* = 528) and right colon tumors (*n* = 419) were most frequent, with fewer rectal cases (*n* = 314). These differences were statistically significant (*p* = 0.02).
- Case distribution by tumor location, patient sex, and age intervals showed RCCs were more common in females under 50 and over 70 years old. In contrast, LCCs and rectal carcinomas were more frequent in males across all age ranges, as shown in Figure 2.
- Regarding *histological type*, ADK (*n* = 1612) was most often found in the left colon (*n* = 680), whereas mucinous ADK (*n* = 191) was more frequently identified in the right colon (*n* = 79). The difference in distribution was statistically significant (Chi-Square test: *p* = 0.01, Fisher’s exact test: *p* = 0.0016). The odds ratio (OR = 0.6102, 95%CI: 0.4520 to 0.8238) indicates that mucinous ADK is less likely to be found in the left colon than in the right colon (see Table 2 and Table 3).
- Mucinous ADK occurred in 43.53% (37/85) of cases in women and 39.62% (42/106) of cases in men, as shown in Figure 3. Other CRC types, such as signet cell carcinoma and medullary carcinoma, were diagnosed in 5 right colon cases and 4 left colon cases.
- For *histological grade*, highly malignant tumors (G3–G4, *n* = 293) were found more often in the right colon (*n* = 141, 24.39%). G1–G2 tumors (*n* = 1519) were observed in similar proportions in the left colon (657 cases—87.48%) and rectum (425 cases—87.99%). The statistical differences were very significant (Chi-Square test: *p* < 0.0001, Fisher’s exact test: *p* < 0.0001, OR = 0.4354, 95%CI: 0.3375 to 0.5617). See Table 2 and Table 3.
- Next, we evaluated the association between tumor location and *pT parameter*. The pT3–pT4 (*n* = 1561) tumors were most common: 518 cases (89.62%) in the right colon, 669 (89.08%) in the left colon, and 374 (77.43%) in the rectum. In contrast, pT1–pT2 tumors (*n* = 251) appeared more in the rectum (109 cases, 22.57%), compared to the left colon (82 cases, 10.92%) and right colon (60 cases, 10.38%). Statistical results: Chi-Square *p* < 0.0001, Fisher’s exact test *p* = 0.0035, OR = 0.6325, 95% CI: 0.4645–0.8613 (see Table 2 and Table 3).
- For regional lymph node status (pN), 916 cases had no metastases (pN0). Metastases occurred in fewer than 4 nodes (pN1) in 492 cases and in more than 4 nodes (pN2) in 404 cases. By tumor location, metastases were more frequently in LCC, with pN1 in 223 cases and pN2 in 153 cases. For RCC, pN1 appeared in 158 cases and pN2 in 130 cases.
- *Pathologically documented metastases* (pM1, *n* = 113) were the most common in RCC patients (*n* = 42).
- Additionally, *lymphovascular invasion* (LVI) was identified more frequently in RCC cases (49.31%) than in LCC cases (46.07%) or rectal carcinomas (45.96%).

## 4. Discussion

The AJCC/UICC TNM staging system, based on the assessment of local tumor extension (T), nodal status (N), and distant metastases (M) [26], remains the “gold standard” for predicting disease progression and therapeutic management of patients with CRC [26,28]. However, some studies demonstrate variability in the clinical outcome among CRC patients at the same stage of disease, thereby diminishing the prognostic and predictive importance of this parameter [29,30,31,32]. Therefore, there remains significant interest in identifying new prognostic and predictive factors to support risk stratification and treatment optimization in CRC patients.

### 4.1. The Relationship Between Tumor Biology and Sidedness

Improved knowledge of molecular biology has led to the anatomical division of the large intestine into the right colon and the left colon, as two independent entities [11,12,13,33]. Usually, tumors that develop on the right part of the colon, up to the level of the splenic flexure, are defined as tumors belonging to the right colon, because the embryonic development of the proximal colon originates from the midgut. In contrast, the distal part originates from the hindgut [16,19,34,35]. There are thus differences between RCC and LCC in terms of origin, exposure to genetic/epigenetic mutations, and the composition of the intestinal flora (microbiota) [36,37,38,39]. The right and left regions of the colon contain distinct bacterial communities, which are thought to influence tumor inflammation and growth, thereby contributing to their unique molecular signatures [5,40,41]. However, the impact of these genetic differences, as well as lifestyle and dietary habits, on carcinogenesis mechanisms, therapeutic response, or prognosis remains largely unknown [40]. The prognostic role of the microbiota in CRC remains under investigation and should not be considered in clinical practice at this time [42,43]. RCC are frequently associated with microsatellite instability–high (MSI-H), BRAF mutations, and the CpG island methylator phenotype (CIMP-high). These molecular features are primarily represented within Consensus Molecular Subtypes (CMS) 1 (immune) and CMS3 (metabolic), both of which are overrepresented in proximal tumors [44,45]. In contrast, LCC more commonly exhibit chromosomal instability with recurrent APC and TP53 mutations and are enriched for the CMS2 (canonical) subtype. This molecular stratification provides a biologically plausible explanation for the more favorable prognosis and increased sensitivity to anti-EGFR–based therapies observed in left-sided tumors [44,45]. Recent advances in single-cell and multi-omics technologies have improved understanding of tumor ecosystems by enabling cell-type–specific analysis. Single-cell RNA sequencing shows that RCC has an immune-enriched microenvironment, with increased infiltration of T lymphocytes and macrophages. Guo et al. reported a higher proportion of exhausted CD8^+^ T cells in RCC, consistent with elevated tumor mutational burden and chronic immune activation [46]. These findings may explain the clinical efficacy of immunotherapy in selected right-sided tumors, especially those with microsatellite instability-high (MSI-H) status. In contrast, LCC shows distinct tumor cell heterogeneity and unique signaling pathway activation. Single-cell profiling has identified specific epithelial cancer cell subpopulations, such as RBP4^+^NTS^+^ cells, that are more common in left-sided tumors and may influence site-specific tumor behavior and metabolism [46]. This cellular specialization may explain the different responses of LCC to conventional cytotoxic and targeted therapies, including anti-EGFR agents, as shown in clinical trials. Therefore, CRC is not a single anatomical entity, and this diversity in tumor location has a key impact on CRC heterogeneity, which is reflected in difficulties in assessing prognosis and response to therapy [13,21,35,40]. Screening strategies and protocols could also be affected by these new findings [43].

### 4.2. The Relationship Between Tumor Location and the Age and Sex of Patients

We included 1812 cases of colorectal carcinoma diagnosed over 10 years in our observational study. The cases were divided into three groups based on location: carcinomas of the right colon (cecum, ascending, and transverse colon)—RCC, carcinomas of the left colon (from the splenic flexure to the sigmoid)—LCC, and rectal carcinomas. LCC cases predominated (41.45%), followed by RCC (31.90%) and rectal carcinomas (26.65%). In a study that analyzed a similar group, it was shown that 68.1% of the patients had tumors located distal to the splenic flexure [47]. We also showed that patients with rectal carcinomas had the lowest mean age at diagnosis, unlike patients with RCC. In patients aged ≤60 years, carcinomas located in the left colon and rectum were more common. In patients >60 years of age, tumors were mainly located in the left and right colon, with statistically significant differences, *p* < 0.0001.

We observed that most colorectal tumors occurred in males (57.95%). This aligns with the current data indicating that CRC affects males more frequently [3,20,48,49]. We observed significant differences between patient sex and tumor anatomical location (*p* < 0.0001). RCCs were relatively evenly distributed across gender categories, with 49.48% of cases diagnosed in females and 50.52% in males, whereas LCCs (59.12%) and rectal carcinomas were more common in males (65.01%). Similar studies report the relationship between the location of the tumor and patient sex. A meta-analysis revealed that more women have proximal colon cancer and that it is frequently diagnosed at an advanced stage [50]. In our study, RCC was more common in young women (under 50 years) and in those over 70 years of age. Despite many studies indicating that older patients are more likely to develop right colon cancer compared to younger age groups [15,51], we only noticed this in women. In these, the increase in the number of cases with age could be related to the disappearance of the protection provided by sex hormones [15,25,52,53]. Other authors have shown that hormone replacement therapy (HRT) is related to initial protection, but the use of HRT after a diagnosis of CRC leads to a more advanced stage of the disease [54]. This may explain the lower 5-year survival rate among women aged 70 or older [55]. Due to the lack of data on HRT use and menopause, we were unable to assess this association in the study group.

### 4.3. The Relationship Between Tumor Location and the Other Histopathological Parameters

The majority of cases were ADK NOS, accounting for 85.47% of RCC, 90.55% of LCC and 90.68% of rectal carcinomas. Mucinous ADK were diagnosed in 10.54% of cases. In the literature, the mucinous histological subtype represents 5% to 20% of CRC cases [56,57,58]. They were identified more frequently in the right colon (41.36% of cases), especially in women (43.53%, 37/85 cases). Most G3-G4 tumors (48.12%) were identified in the right colon (*p* < 0.0001). RCCs more frequently showed infiltrative aspects (*p* < 0.0001) and distant metastases (in 37.17% of cases), with results similar to those of other specialized studies [59]. Other authors have also noted that patients with proximal colon cancers had a higher tumor grade compared to patients with sigmoid colon cancer, who had lower-grade tumors and a better prognosis [60]. In our study, early-stage ADK NOS (pT1-T2) were diagnosed almost twice as often in the rectum as in the right colon. G1-G2 cases also predominated in the distal colon. In another study, we demonstrated that the location of CRC was statistically significantly correlated with pT parameter and with the tumor board configuration of infiltrative type [31]. The literature has described the correlation between tumor location and the depth of tumor infiltration into the wall. The rectal tumors, followed by LCC, discovered at an early stage, are most likely related to symptoms and the use of colonoscopy [43,61]. Other authors have also noted that sigmoid colon cancers were detected more often at stage I compared to cancers with more proximal locations [62]. They have also shown a relative decrease in LCC incidence and a gradual increase in RCC, especially for primary cecal neoplasms [62]. This variation in the anatomical distribution of CRC may be partly due to screening, as screening methods (colonoscopy/occult blood testing) are more effective/sensitive for detecting tumors on the left side of the colon [49,62]. Furthermore, chromosomal instability, K-RAS mutation, and EGFR or HER2 overexpression are more commonly observed in LCC [40]. On the other hand, RCCs are more commonly diagnosed in women and are sometimes mucinous ADK, with high-grade microsatellite instability (MSI), CpG island methylation phenotype, and BRAF mutation [40,63].

### 4.4. The Importance of Tumor Location on Treatment and Prognosis

It has been observed that tumor location correlates with disease progression and is an important factor in risk stratification of patients with CRC [12,60]. Yahagi et al. demonstrated that RCC patients have a poorer prognosis than those with LCC [14]. RCC tumors are primarily driven by MSI-high status, CIMP-high phenotypes, and BRAF mutations, which often correlate with a more aggressive clinical course and poorer survival [5,40]. LCCs typically exhibit chromosomal instability and TP53 mutations, a molecular profile that directly predicts superior outcomes and heightened sensitivity to anti-EGFR targeted therapies [5,40]. Sidedness acts as a surrogate for these molecular drivers, allowing clinicians to tailor treatment by choosing immunotherapy for MSI-high proximal cases or EGFR inhibitors for RAS wild-type distal cases [5,34,40]. In another meta-analysis, patients with metastatic CRC resulting from RCC have a lower survival rate than patients with LCC [47]. According to other authors, tumor location influences the success of systemic treatment and the progression of metastatic CRC [59,64]. Thus, RCC has a more unfavorable prognosis than LCC, as patients present at a more advanced stage and the rate of postoperative complications is higher [64]. Treatment is also different, with neoadjuvant radiotherapy playing a predominant role in rectal carcinomas [59]. As suggested by Şirin et al., the survival rate of patients with rectal cancer decreases inversely proportional to the tumoral extension [65]. Another study showed that the risk of recurrence is higher in the first few years after initial diagnosis, even among young patients [66]. Patients with recurrent cancer were more often elderly, female, and those who had a less differentiated initial tumor in the proximal colon [66]. Other authors have reported lower survival rates among patients with RCC than among those with LCC [67]. Moreover, it was demonstrated that, after chemotherapy treatment, the outcome of LCC patients was better compared to RCC patients for patients with metastatic CRC [68]. Petrelli et al. showed that tumor topography is an important prognostic parameter in patients with early CRC and metastatic disease, with LCC being associated with a lower mortality rate [34]. In addition, topography is also a strong predictor of patient outcome after antiEGFR treatment in wild-type RAS metastatic CRC patients [34]. The survival of RCC patients is generally limited when Cetuximab or Panitumumab is added to chemotherapy [69]. Another study demonstrated that primary tumor location is an independent prognostic factor in both locally advanced and metastatic CRC patients, with LCC patients having a better overall survival (OS) and median disease-free survival (DFS) [70].

The molecular and single-cell findings complement the clinical observations described in this manuscript and support that CRC sidedness is biologically determined. The integration of clinicopathological features with OMICS data enhances the interpretation of prognosis and treatment response, underscoring tumor location as a critical factor in colorectal cancer management.

### 4.5. Study Limitations

Our analysis is subject to several constraints, primarily stemming from the lack of a centralized national oncology registry and of data on the postoperative evolution of patients. These systemic limitations restricted our ability to monitor long-term patient outcomes and therapeutic responses, and to provide a definitive assessment of disease incidence and mortality at a population level. Additionally, the retrospective nature of this single-center study, coupled with insufficient data regarding familial predispositions and environmental risk factors, may affect the generalizability of the results. Moreover, we excluded cases that received neoadjuvant therapy to have an accurate assessment of the original histopathological features. The following histological changes may occur after neoadjuvant treatment: disappearance or reduction in tumor/regional lymph node volume, morphological changes such as fibrosis and inflammation, difficulty in establishing histological grade, cellular regression (cytonuclear pleomorphism, cytoplasmic vacuolization), acellular mucin that may be misinterpreted as a mucinous component or mucinous carcinoma, disappearance of lymphovascular invasion, and perineural invasion. Tumor regression introduced by treatment will modify the real biological differences between RCC, LCC, and rectal carcinomas. Including these cases will not reflect the tumor’s true aggressiveness at the time of diagnosis. Nevertheless, we believe the substantial cohort size and the extensive timeframe analyzed offer a robust and meaningful perspective on the pathological trends within our institution. We have demonstrated clinicopathological differences between RCC, LCC, and rectal carcinomas, and the significance of tumor location as a risk stratification parameter. Still, the impact of primary tumor location on the prognosis of patients with CRC remains to be further investigated.

## 5. Conclusions

Clinicopathological characteristics of tumors varied according to the primary tumor site. LCC and rectal tumors were more prevalent in men. RCC was more frequently identified in women under 50 years and over 70 years of age. Early invasive tumors were predominantly rectal carcinomas, while tumors with advanced wall extension were primarily RCC. Additionally, RCC was associated with distant metastases and a mucinous tumor phenotype. Other histological types with aggressive prognoses were also more commonly diagnosed in the RCC category. Collectively, these findings support the risk stratification of CRC patients by tumor location in both retrospective and prospective analyses, which may inform therapeutic strategies and prognosis evaluation across all disease stages. Thus, primary tumor location should be considered when determining the need for personalized systemic therapeutic approaches and screening programs in CRC patients.

## Figures and Tables

**Table 1:** The clinicopathological characteristics of patients with colorectal carcinomas.

Parameters	No Cases (*n* = 1812)	Percentage (%)
**Females**	762	42.05
**Males**	1050	57.95
**≤60 years**	551	30.41
**>60 years**	1261	69.59
**RCC**	578	31.90
**LCC**	751	41.45
**Rectal tumors**	483	26.66
**ADK NOS**	1612	88.96
**Mucinous ADK**	191	10.54
**Others types**	9	0.50
**G1–G2**	1519	83.83
**G3–G4**	293	16.17
**pT1**	37	2.04
**pT2**	214	11.81
**pT3**	991	54.69
**pT4**	570	31.46
**pN0**	916	50.55
**pN1**	492	27.15
**pN2**	404	22.30
**Mx**	1700	93.82
**pM1**	112	6.18
**LV0**	959	52.92
**LV1**	853	47.08

RCC—right colon carcinomas. LCC—left colon carcinomas. ADK—adenocarcinoma. ADK NOS—classical/conventional type adenocarcinoma (Not Otherwise Specified). G1–G2—low degree of malignancy. G3–G4—high degree of malignancy. pT1—tumor extension into the submucosa, pT2–tumor extension into the muscularis propria. pT3—tumor extension into the subserosa/pericolic adipose tissue. pT4—tumor extension into or through the serosa. pN0—absence of nodal metastases. pN1—1 to 3 positive lymph nodes. pN2—more than three lymph nodes with metastases. Mx—unknown distant metastases. pM1—presence of distant metastases. LV0—absence of lymphovascular invasion. LV1—presence of lymphovascular invasion.

**Table 2:** The relationship between tumor location and other prognostic parameters was analyzed using the Chi-square test.

Parameters		RCC (%)	LCC (%)	Rectal Carcinomas (%)	Chi-Square Test
**Total cases (%)**		578 (31.90)	751 (41.45)	483 (26.66)	***p* value**
**Sex**	Females	286 (49.48)	307 (40.88)	169 (34.99)	<0.0001
	Males	292 (50.52)	444 (59.12)	314 (65.01)	
**Age of diagnosis**	≤60 years	159 (27.51)	223 (29.69)	169 (34.99)	0.02
	>60 years	419 (72.49)	528 (70.31)	314 (65.01)	
**Histological subtypes**	ADK NOS	494 (85.47)	680 (90.55)	438 (90.68)	0.01
	Mucinous ADK	79 (13.67)	67 (8.92)	45 (9.32)	
	Others types	5 (0.87)	4 (0.53)	0	
**Histological grade of differentiation**	G1–G2	437 (75.61)	657 (87.48)	425 (87.99)	<0.0001
	G3–G4	141 (24.39)	94 (12.52)	58 (12.01)	
**Depth of tumor invasion**	pT1	8 (1.38)	16 (2.13)	13 (2.69)	<0.0001
	pT2	52 (9.00)	66 (8.79)	96 (19.88)	
	pT3	312 (53.98)	409 (54.46)	270 (55.90)	
	pT4	206 (35.64)	260 (34.62)	104 (21.53)	
**Lymph node status**	pN0	290 (50.17)	375 (49.93)	251 (51.97)	0.08
	pN1	158 (27.34)	223 (29.69)	111 (22.98)	
	pN2	130 (22.49)	153 (20.37)	121 (25.05)	
**Distant metastases**	Mx	536 (92.73)	712 (94.81)	451 (93.37)	0.27
	pM1	42 (7.27)	39 (5.19)	32 (6.63)	
**Lymphovascular invasion**	LV0	293 (50.69)	405 (53.93)	26 (54.04)	0.42
	LV1	285 (49.31)	346 (46.07)	222 (45.96)	

RCC—right colon carcinomas. LCC—left colon carcinomas. ADK—adenocarcinoma. ADK NOS—classical/conventional type adenocarcinoma (Not Otherwise Specified). G1–G2—low degree of malignancy. G3–G4—high degree of malignancy. pT1—tumor extension into the submucosa, pT2—tumor extension into the muscularis propria. pT3—tumor extension into the subserosa/pericolic adipose tissue. pT4—tumor extension into or through the serosa. pN0—absence of nodal metastases. pN1—1 to 3 positive lymph nodes. pN2—more than three lymph nodes with metastases. Mx—unknown distant metastases. pM1—presence of distant metastases. LV0—absence of lymphovascular invasion. LV1—presence of lymphovascular invasion.

**Table 3:** Statistical analysis of correlations between tumor location and other prognostic parameters, using the two-sided Fisher’s exact test.

Parameters	Proximal Tumors (RCC)	Distal Tumors (LCC and Rectal Carcinomas)	Fisher’s Exact Test		
**No cases**	578	1234	***p* value**	**Odds ratio**	**95% confidence interval**
**Females**	286	476	<0.0001	1.56	1.278 to 1.904
**Males**	292	758			
**≤60 years**	159	392	0.0706	0.8151	0.6552 to 1.014
**>60 years**	419	842			
**ADK NOS**	494	1118	0.0016	0.6102	0.4520 to 0.8238
**Nonmucinous ADK**	84	116			
**G1–G2**	437	1082	<0.0001	0.4354	0.3375 to 0.5617
**G3–G4**	141	152			
**pT1–pT2**	60	191	0.0035	0.6325	0.4645 to 0.8613
**pT3–pT4**	518	1043			
**pN0**	290	626	0.8403	0.978	0.8026 to 1.192
**pN1-pN2**	288	608			
**Mx**	536	1164	0.209	0.7675	0.5165 to 1.140
**pM1**	42	70			
**LV0**	293	666	0.2069	0.8768	0.7194 to 1.069
**LV1**	285	568			

RCC—right colon carcinomas. LCC—left colon carcinomas. ADK—adenocarcinoma. ADK NOS—classical/conventional type adenocarcinoma (Not Otherwise Specified). G1–G2—low degree of malignancy. G3–G4—high degree of malignancy. pT1—tumor extension into the submucosa, pT2—tumor extension into the muscularis propria. pT3—tumor extension into the subserosa/pericolic adipose tissue. pT4—tumor extension into or through the serosa. pN0—absence of nodal metastases. pN1—1 to 3 positive lymph nodes. pN2—more than three lymph nodes with metastases. Mx—unknown distant metastases. pM1—presence of distant metastases. LV0—absence of lymphovascular invasion. LV1—presence of lymphovascular invasion.

## Data Availability

Data generated or analyzed during this study are included in this published article and can be provided if needed or requested by the reviewer.

## Abbreviations

The following abbreviations are used in this manuscript:

CRC	Colorectal cancer
RCC	Right colon carcinomas
LCC	Left colon carcinomas
PBCCEHT	“Pius Brânzeu” County Clinical Emergency Hospital Timișoara
MSI	microsatellite instability
HE	Hematoxylin-eosin
WHO	World Health Organization
AJCC	American Joint Committee on Cancer
UICC	International Union for Cancer Control
pT	Depth of tumor invasion into the intestinal wall
pN	Lymph node status
pM	Distant metastases
LVI	Lymphovascular invasion
F	Female
M	Male
ADK	Adenocarcinoma
ADK NOS	Classic, conventional type adenocarcinoma
*n*	Number of cases
HRT	Hormone replacement therapy
